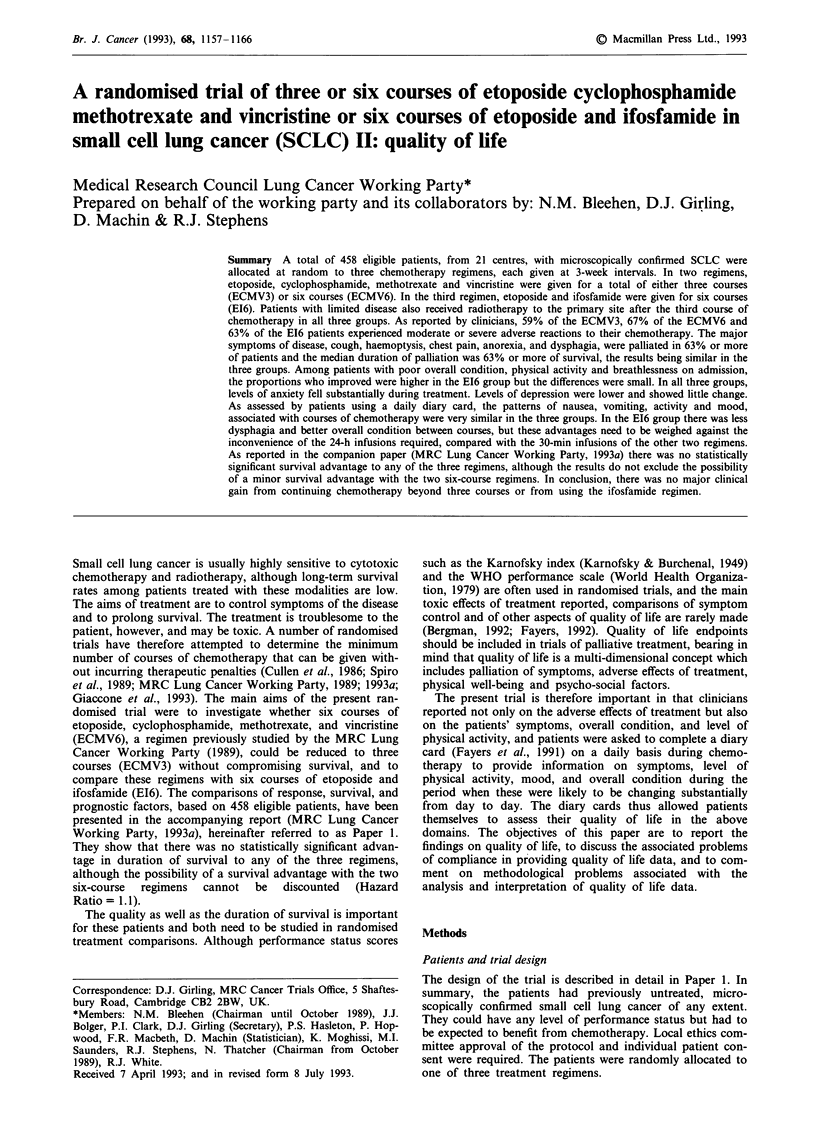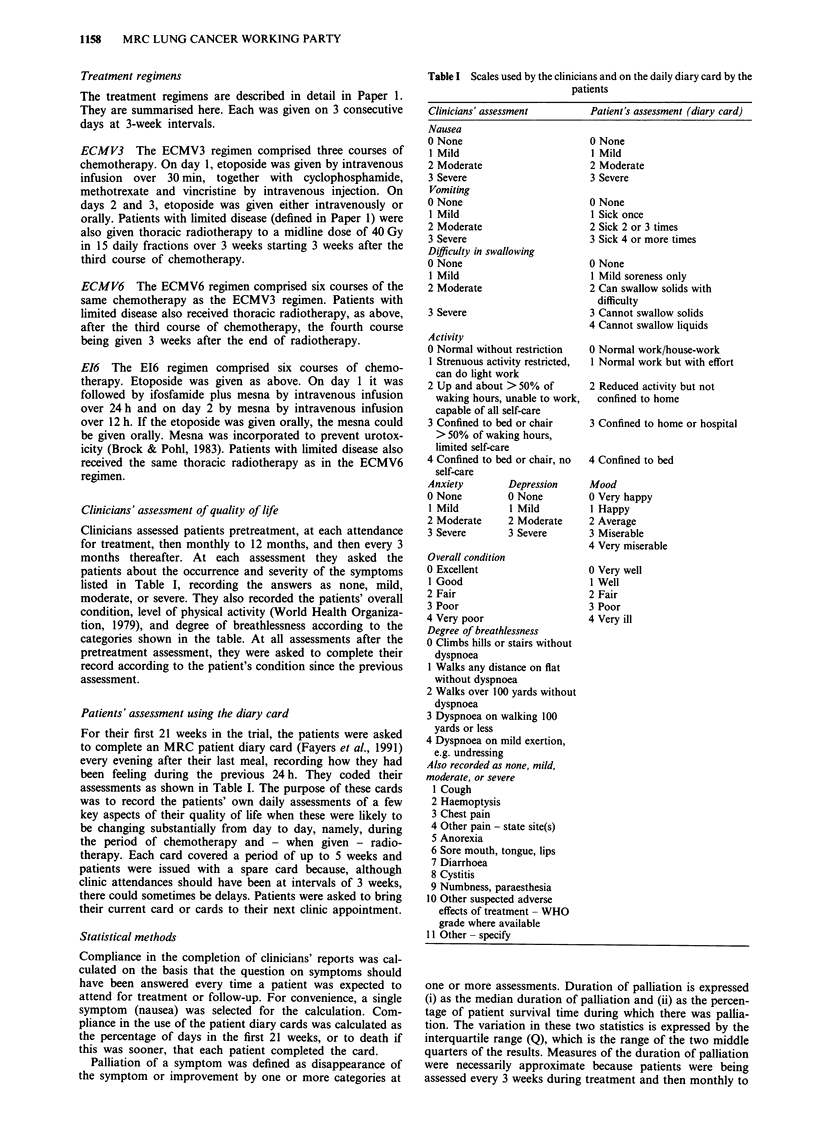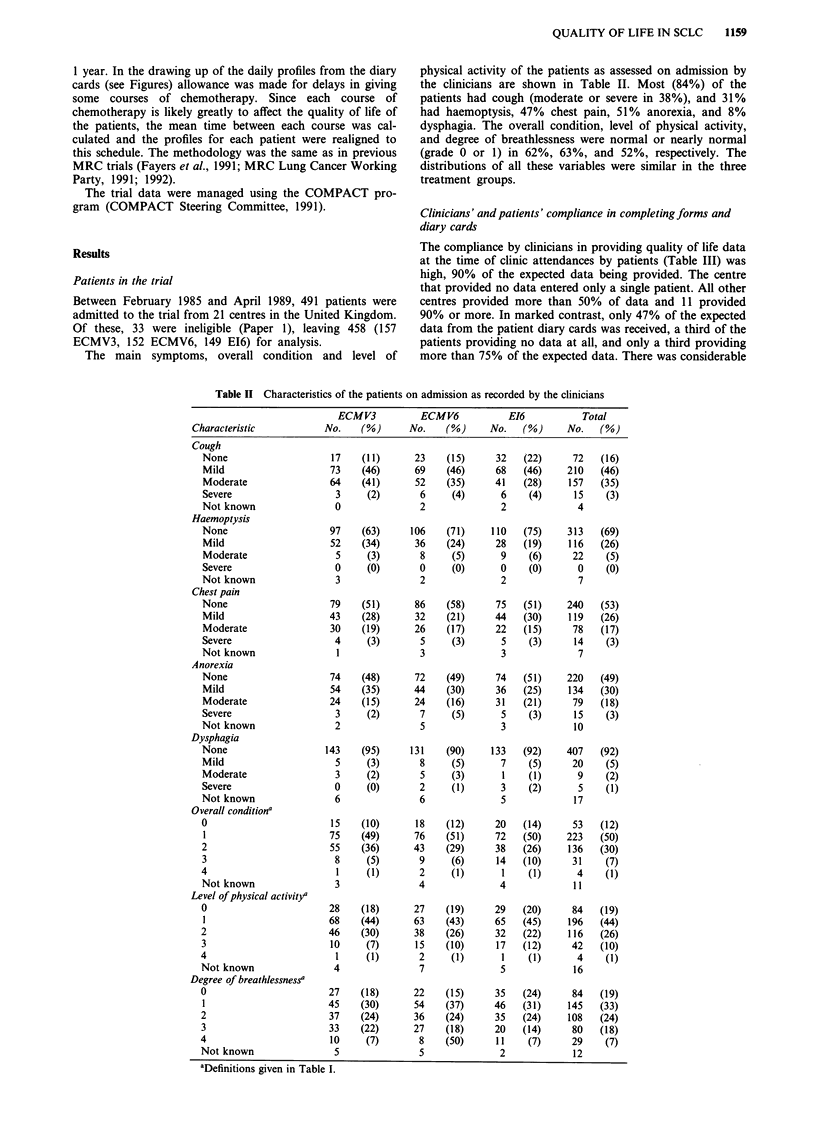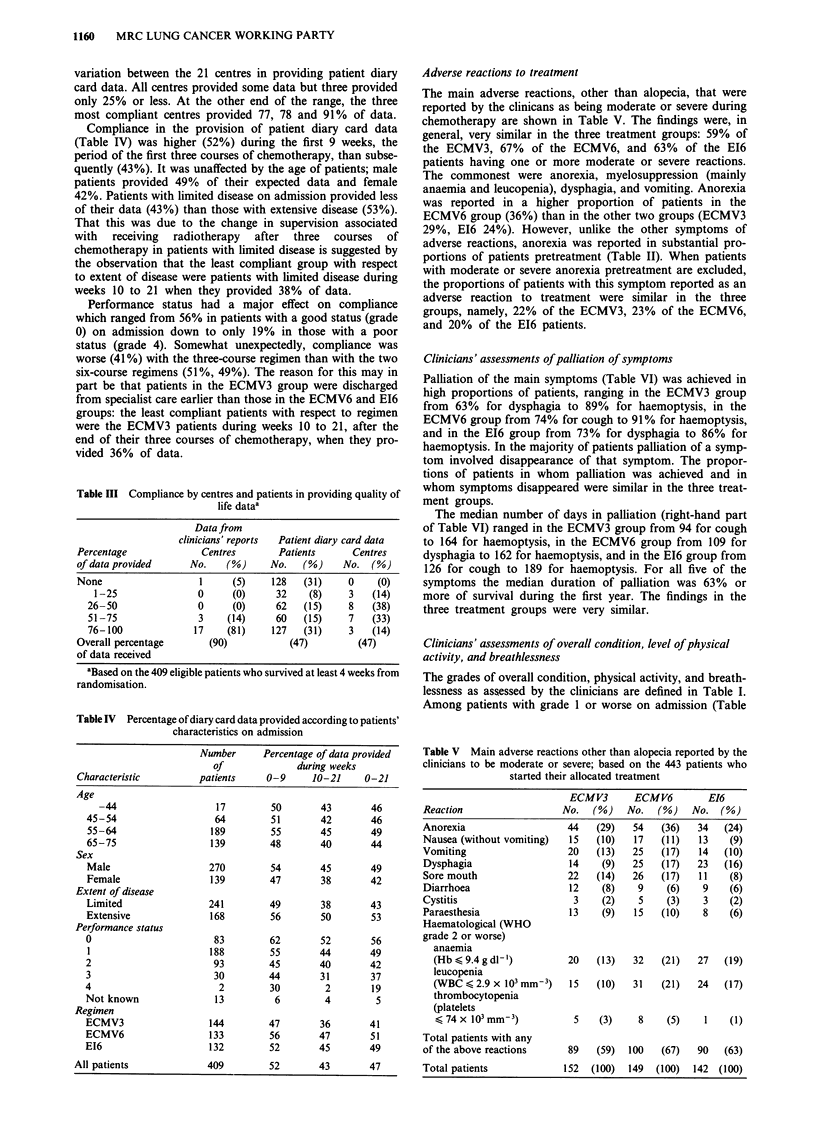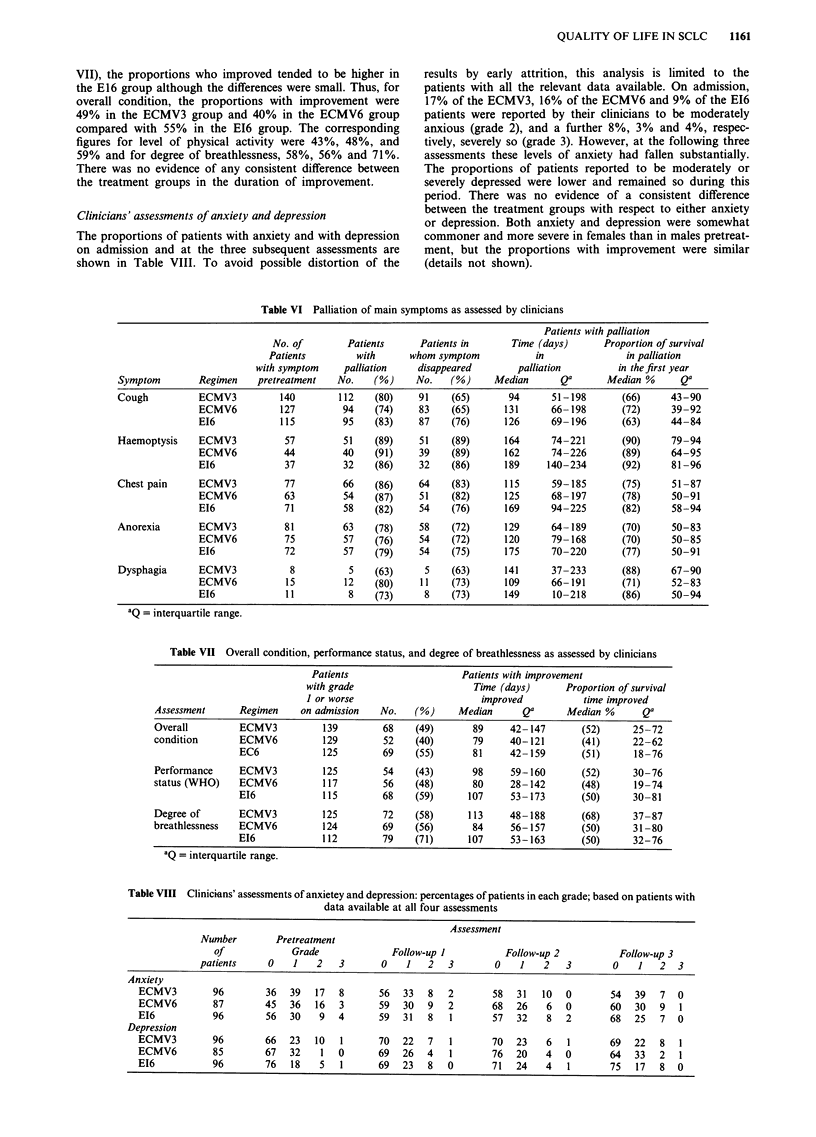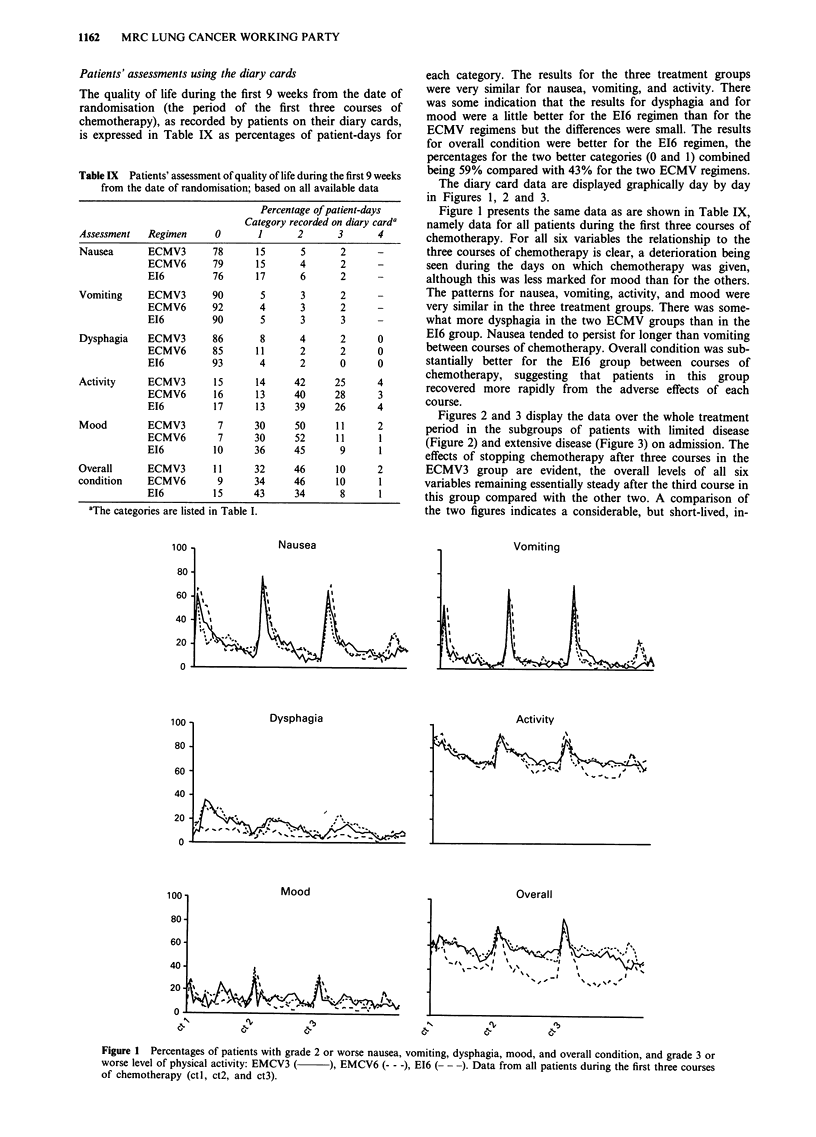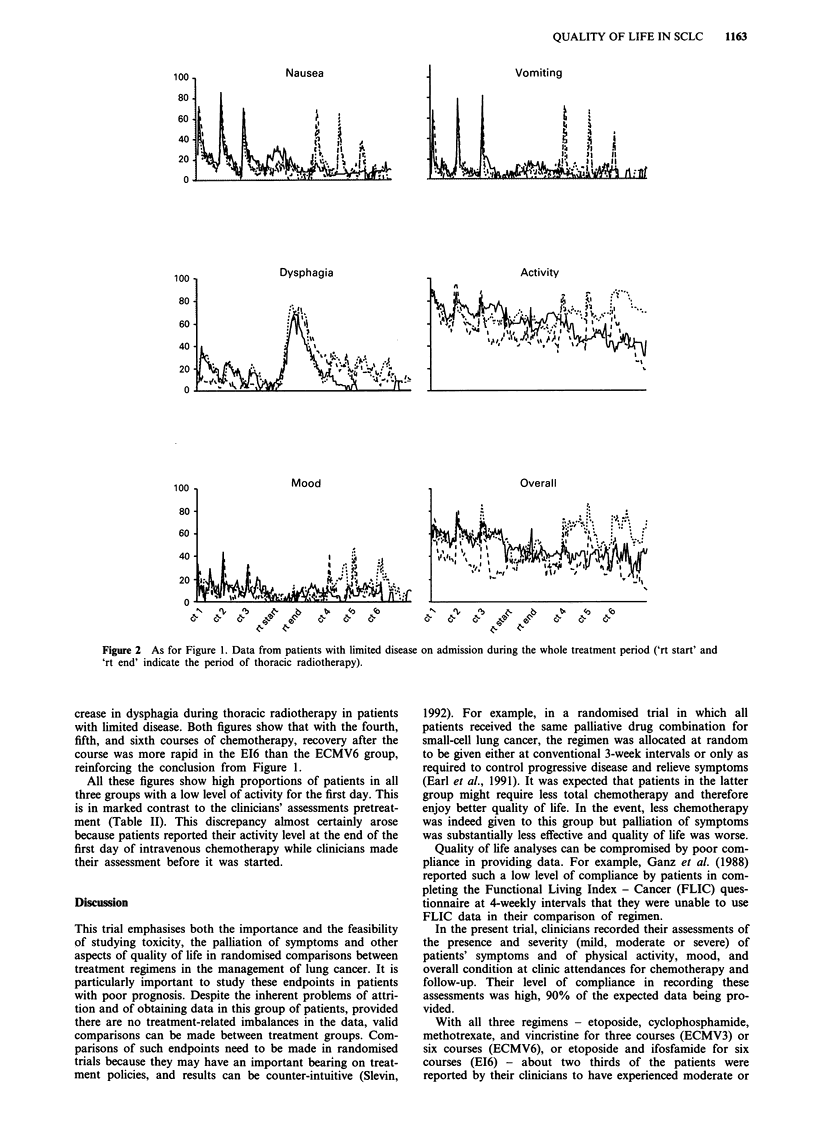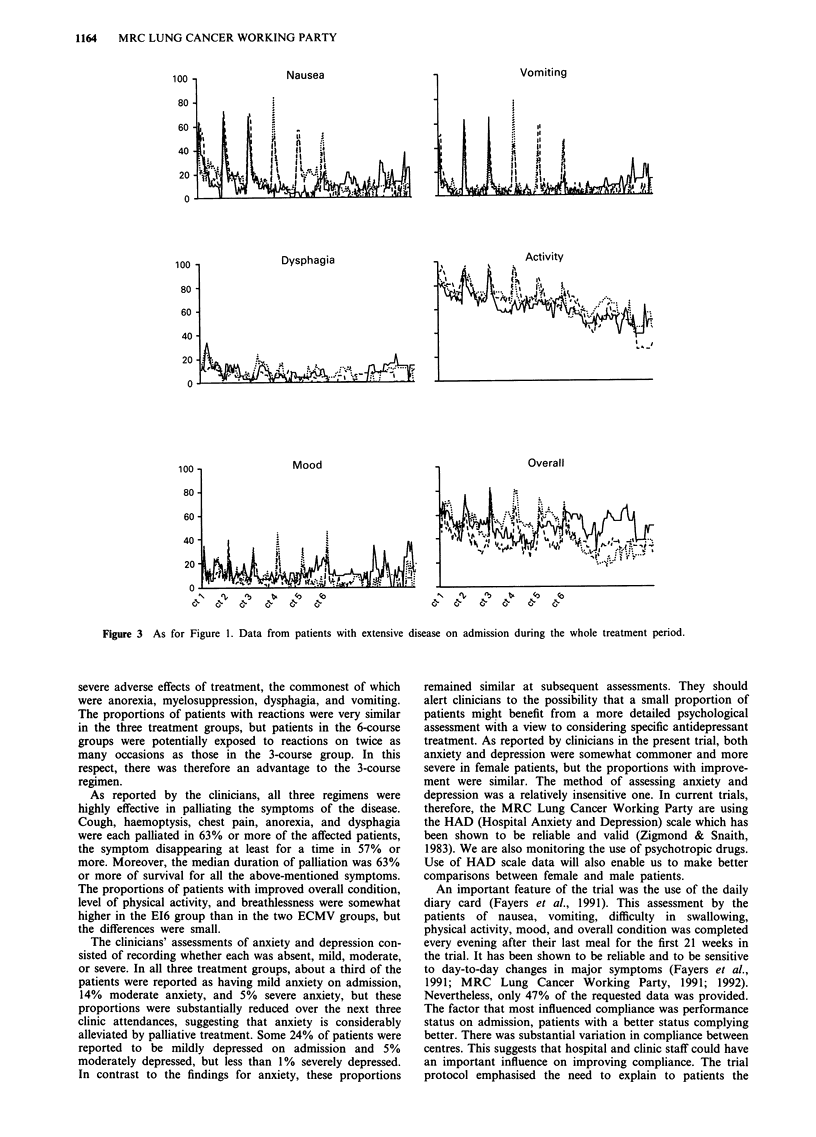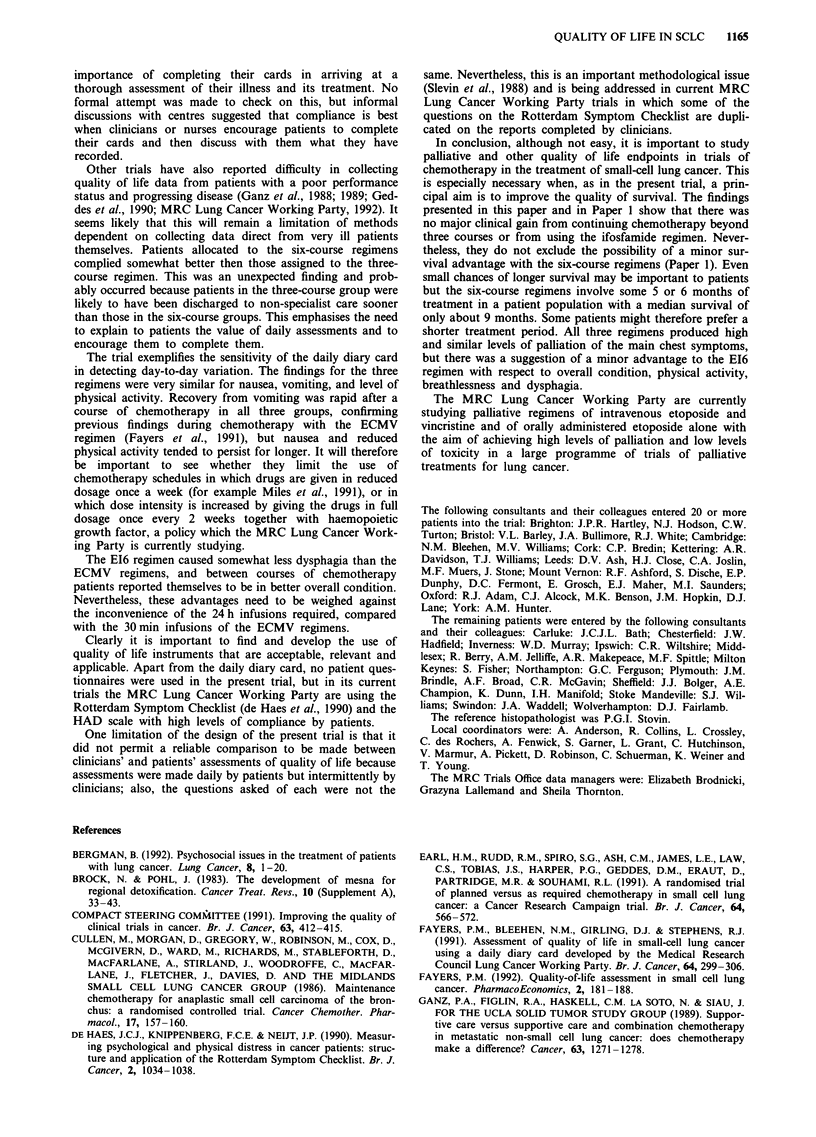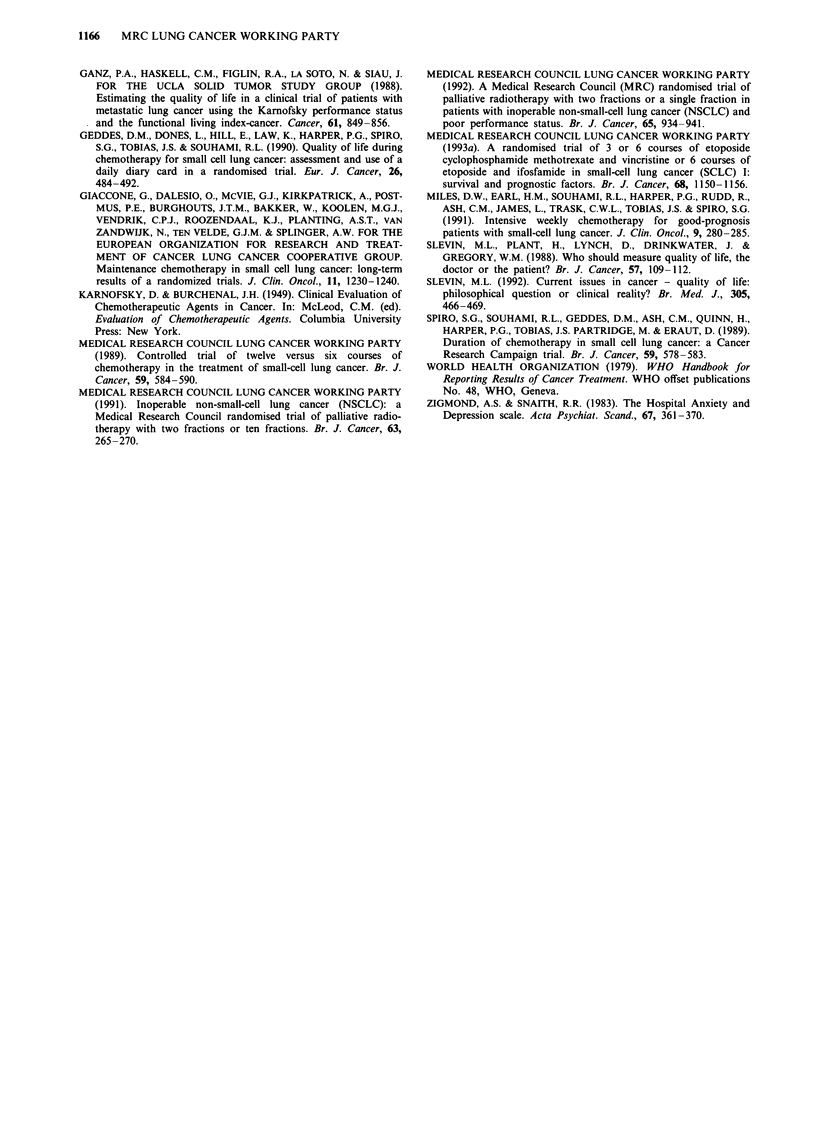# A randomised trial of three or six courses of etoposide cyclophosphamide methotrexate and vincristine or six courses of etoposide and ifosfamide in small cell lung cancer (SCLC). II: Quality of life. Medical Research Council Lung Cancer Working Party.

**DOI:** 10.1038/bjc.1993.497

**Published:** 1993-12

**Authors:** N. M. Bleehen, D. J. Girling, D. Machin, R. J. Stephens

## Abstract

A total of 458 eligible patients, from 21 centres, with microscopically confirmed SCLC were allocated at random to three chemotherapy regimens, each given at 3-week intervals. In two regimens, etoposide, cyclophosphamide, methotrexate and vincristine were given for a total of either three courses (ECMV3) or six courses (ECMV6). In the third regimen, etoposide and ifosfamide were given for six courses (E16). Patients with limited disease also received radiotherapy to the primary site after the third course of chemotherapy in all three groups. As reported by clinicians, 59% of the ECMV3, 67% of the ECMV6 and 63% of the EI6 patients experienced moderate or severe adverse reactions to their chemotherapy. The major symptoms of disease, cough, haemoptysis, chest pain, anorexia, and dysphagia, were palliated in 63% or more of patients and the median duration of palliation was 63% or more of survival, the results being similar in the three groups. Among patients with poor overall condition, physical activity and breathlessness on admission, the proportions who improved were higher in the EI6 group but the differences were small. In all three groups, levels of anxiety fell substantially during treatment. Levels of depression were lower and showed little change. As assessed by patients using a daily diary card, the patterns of nausea, vomiting, activity and mood, associated with courses of chemotherapy were very similar in the three groups. In the EI6 group there was less dysphagia and better overall condition between courses, but these advantages need to be weighed against the inconvenience of the 24-h infusions required, compared with the 30-min infusions of the other two regimens. As reported in the companion paper (MRC Lung Cancer Working Party, 1993a) there was no statistically significant survival advantage to any of the three regimens, although the results do not exclude the possibility of a minor survival advantage with the two six-course regimens. In conclusion, there was no major clinical gain from continuing chemotherapy beyond three courses or from using the ifosfamide regimen.


Br. J. Cancer (1993), 68, 1157-1166 © Macmillan Press Ltd., 1993

### A randomised trial of three or six courses of etoposide cyclophosphamide methotrexate and vincristine or six courses of etoposide and ifosfamide in small cell lung cancer (SCLC) II: quality of life

Medical Research Council Lung Cancer Working Party*
Prepared on behalf of the working party and its collaborators by: N.M. Bleehen, D.J. Girling, D. Machin & R.J. Stephens

**Summary** A total of 458 eligible patients, from 21 centres, with microscopically confirmed SCLC were allocated at random to three chemotherapy regimens, each given at 3-week intervals. In two regimens, etoposide, cyclophosphamide, methotrexate and vincristine were given for a total of either three courses (ECMV3) or six courses (ECMV6). In the third regimen, etoposide and ifosfamide were given for six courses (EI6). Patients with limited disease also received radiotherapy to the primary site after the third course of chemotherapy in all three groups. As reported by clinicians, 59% of the ECMV3, 67% of the ECMV6 and 63% of the EI6 patients experienced moderate or severe adverse reactions to their chemotherapy. The major symptoms of disease, cough, haemoptysis, chest pain, anorexia, and dysphagia, were palliated in 63% or more of patients and the median duration of palliation was 63% or more of survival, the results being similar in the three groups. Among patients with poor overall condition, physical activity and breathlessness on admission, the proportions who improved were higher in the EI6 group but the differences were small. In all three groups, levels of anxiety fell substantially during treatment. Levels of depression were lower and showed little change. As assessed by patients using a daily diary card, the patterns of nausea, vomiting, activity and mood, associated with courses of chemotherapy were very similar in the three groups. In the EI6 group there was less dysphagia and better overall condition between courses, but these advantages need to be weighed against the inconvenience of the 24-h infusions required, compared with the 30-min infusions of the other two regimens. As reported in the companion paper (MRC Lung Cancer Working Party, 1993*a*) there was no statistically significant survival advantage to any of the three regimens, although the results do not exclude the possibility of a minor survival advantage with the two six-course regimens. In conclusion, there was no major clinical gain from continuing chemotherapy beyond three courses or from using the ifosfamide regimen.

Small cell lung cancer is usually highly sensitive to cytotoxic chemotherapy and radiotherapy, although long-term survival rates among patients treated with these modalities are low. The aims of treatment are to control symptoms of the disease and to prolong survival. The treatment is troublesome to the patient, however, and may be toxic. A number of randomised trials have therefore attempted to determine the minimum number of courses of chemotherapy that can be given without incurring therapeutic penalties (Cullen *et al.*, 1986; Spiro *et al.*, 1989; MRC Lung Cancer Working Party, 1989; 1993*a*; Giaccone *et al.*, 1993). The main aims of the present randomised trial were to investigate whether six courses of etoposide, cyclophosphamide, methotrexate, and vincristine (ECMV6), a regimen previously studied by the MRC Lung Cancer Working Party (1989), could be reduced to three courses (ECMV3) without compromising survival, and to compare these regimens with six courses of etoposide and ifosfamide (EI6). The comparisons of response, survival, and prognostic factors, based on 458 eligible patients, have been presented in the accompanying report (MRC Lung Cancer Working Party, 1993*a*), hereinafter referred to as Paper 1. They show that there was no statistically significant advantage in duration of survival to any of the three regimens, although the possibility of a survival advantage with the two six-course regimens cannot be discounted (Hazard Ratio = 1.1).

The quality as well as the duration of survival is important for these patients and both need to be studied in randomised treatment comparisons. Although performance status scores such as the Karnofsky index (Karnofsky & Burchenal, 1949) and the WHO performance scale (World Health Organization, 1979) are often used in randomised trials, and the main toxic effects of treatment reported, comparisons of symptom control and of other aspects of quality of life are rarely made (Bergman, 1992; Fayers, 1992). Quality of life endpoints should be included in trials of palliative treatment, bearing in mind that quality of life is a multi-dimensional concept which includes palliation of symptoms, adverse effects of treatment, physical well-being and psycho-social factors.

The present trial is therefore important in that clinicians reported not only on the adverse effects of treatment but also on the patients' symptoms, overall condition, and level of physical activity, and patients were asked to complete a diary card (Fayers *et al.*, 1991) on a daily basis during chemotherapy to provide information on symptoms, level of physical activity, mood, and overall condition during the period when these were likely to be changing substantially from day to day. The diary cards thus allowed patients themselves to assess their quality of life in the above domains. The objectives of this paper are to report the findings on quality of life, to discuss the associated problems of compliance in providing quality of life data, and to comment on methodological problems associated with the analysis and interpretation of quality of life data.

### Methods

### *Patients and trial design*

The design of the trial is described in detail in Paper 1. In summary, the patients had previously untreated, microscopically confirmed small cell lung cancer of any extent. They could have any level of performance status but had to be expected to benefit from chemotherapy. Local ethics committee approval of the protocol and individual patient consent were required. The patients were randomly allocated to one of three treatment regimens.

Correspondence: D.J. Girling, MRC Cancer Trials Office, 5 Shaftesbury Road, Cambridge CB2 2BW, UK.
*Members: N.M. Bleehen (Chairman until October 1989), J.J. Bolger, P.I. Clark, D.J. Girling (Secretary), P.S. Hasleton, P. Hopwood, F.R. Macbeth, D. Machin (Statistician), K. Moghissi, M.I. Saunders, R.J. Stephens, N. Thatcher (Chairman from October 1989), R.J. White.
Received 7 April 1993; and in revised form 8 July 1993.

*Treatment regimens*

The treatment regimens are described in detail in Paper 1. They are summarised here. Each was given on 3 consecutive days at 3-week intervals.

*ECMV3* The ECMV3 regimen comprised three courses of chemotherapy. On day 1, etoposide was given by intravenous infusion over 30 min, together with cyclophosphamide, methotrexate and vincristine by intravenous injection. On days 2 and 3, etoposide was given either intravenously or orally. Patients with limited disease (defined in Paper 1) were also given thoracic radiotherapy to a midline dose of 40 Gy in 15 daily fractions over 3 weeks starting 3 weeks after the third course of chemotherapy.

*ECMV6* The ECMV6 regimen comprised six courses of the same chemotherapy as the ECMV3 regimen. Patients with limited disease also received thoracic radiotherapy, as above, after the third course of chemotherapy, the fourth course being given 3 weeks after the end of radiotherapy.

*EI6* The EI6 regimen comprised six courses of chemotherapy. Etoposide was given as above. On day 1 it was followed by ifosfamide plus mesna by intravenous infusion over 24 h and on day 2 by mesna by intravenous infusion over 12 h. If the etoposide was given orally, the mesna could be given orally. Mesna was incorporated to prevent urotoxicity (Brock & Pohl, 1983). Patients with limited disease also received the same thoracic radiotherapy as in the ECMV6 regimen.

*Clinicians' assessment of quality of life*

Clinicians assessed patients pretreatment, at each attendance for treatment, then monthly to 12 months, and then every 3 months thereafter. At each assessment they asked the patients about the occurrence and severity of the symptoms listed in Table I, recording the answers as none, mild, moderate, or severe. They also recorded the patients' overall condition, level of physical activity (World Health Organization, 1979), and degree of breathlessness according to the categories shown in the table. At all assessments after the pretreatment assessment, they were asked to complete their record according to the patient's condition since the previous assessment.

*Patients' assessment using the diary card*

For their first 21 weeks in the trial, the patients were asked to complete an MRC patient diary card (Fayers *et al.*, 1991) every evening after their last meal, recording how they had been feeling during the previous 24 h. They coded their assessments as shown in Table I. The purpose of these cards was to record the patients' own daily assessments of a few key aspects of their quality of life when these were likely to be changing substantially from day to day, namely, during the period of chemotherapy and – when given – radiotherapy. Each card covered a period of up to 5 weeks and patients were issued with a spare card because, although clinic attendances should have been at intervals of 3 weeks, there could sometimes be delays. Patients were asked to bring their current card or cards to their next clinic appointment.

*Statistical methods*

Compliance in the completion of clinicians' reports was calculated on the basis that the question on symptoms should have been answered every time a patient was expected to attend for treatment or follow-up. For convenience, a single symptom (nausea) was selected for the calculation. Compliance in the use of the patient diary cards was calculated as the percentage of days in the first 21 weeks, or to death if this was sooner, that each patient completed the card.

Palliation of a symptom was defined as disappearance of the symptom or improvement by one or more categories at one or more assessments. Duration of palliation is expressed (i) as the median duration of palliation and (ii) as the percentage of patient survival time during which there was palliation. The variation in these two statistics is expressed by the interquartile range (Q), which is the range of the two middle quarters of the results. Measures of the duration of palliation were necessarily approximate because patients were being assessed every 3 weeks during treatment and then monthly to

**Table I** Scales used by the clinicians and on the daily diary card by the patients

| *Clinicians' assessment* | | *Patient's assessment (diary card)* |
|---|---|---|
| *Nausea* | | |
| 0 None | | 0 None |
| 1 Mild | | 1 Mild |
| 2 Moderate | | 2 Moderate |
| 3 Severe | | 3 Severe |
| *Vomiting* | | |
| 0 None | | 0 None |
| 1 Mild | | 1 Sick once |
| 2 Moderate | | 2 Sick 2 or 3 times |
| 3 Severe | | 3 Sick 4 or more times |
| *Difficulty in swallowing* | | |
| 0 None | | 0 None |
| 1 Mild | | 1 Mild soreness only |
| 2 Moderate | | 2 Can swallow solids with difficulty |
| 3 Severe | | 3 Cannot swallow solids |
| | | 4 Cannot swallow liquids |
| *Activity* | | |
| 0 Normal without restriction | | 0 Normal work/house-work |
| 1 Strenuous activity restricted, can do light work | | 1 Normal work but with effort |
| 2 Up and about >50% of waking hours, unable to work, capable of all self-care | | 2 Reduced activity but not confined to home |
| 3 Confined to bed or chair >50% of waking hours, limited self-care | | 3 Confined to home or hospital |
| 4 Confined to bed or chair, no self-care | | 4 Confined to bed |
| *Anxiety* | *Depression* | *Mood* |
| 0 None | 0 None | 0 Very happy |
| 1 Mild | 1 Mild | 1 Happy |
| 2 Moderate | 2 Moderate | 2 Average |
| 3 Severe | 3 Severe | 3 Miserable |
| | | 4 Very miserable |
| *Overall condition* | | |
| 0 Excellent | | 0 Very well |
| 1 Good | | 1 Well |
| 2 Fair | | 2 Fair |
| 3 Poor | | 3 Poor |
| 4 Very poor | | 4 Very ill |
| *Degree of breathlessness* | | |
| 0 Climbs hills or stairs without dyspnoea | | |
| 1 Walks any distance on flat without dyspnoea | | |
| 2 Walks over 100 yards without dyspnoea | | |
| 3 Dyspnoea on walking 100 yards or less | | |
| 4 Dyspnoea on mild exertion, e.g. undressing | | |
| *Also recorded as none, mild, moderate, or severe* | | |
| 1 Cough | | |
| 2 Haemoptysis | | |
| 3 Chest pain | | |
| 4 Other pain – state site(s) | | |
| 5 Anorexia | | |
| 6 Sore mouth, tongue, lips | | |
| 7 Diarrhoea | | |
| 8 Cystitis | | |
| 9 Numbness, paraesthesia | | |
| 10 Other suspected adverse effects of treatment – WHO grade where available | | |
| 11 Other – specify | | |

1 year. In the drawing up of the daily profiles from the diary cards (see Figures) allowance was made for delays in giving some courses of chemotherapy. Since each course of chemotherapy is likely greatly to affect the quality of life of the patients, the mean time between each course was calculated and the profiles for each patient were realigned to this schedule. The methodology was the same as in previous MRC trials (Fayers *et al.*, 1991; MRC Lung Cancer Working Party, 1991; 1992).

The trial data were managed using the COMPACT program (COMPACT Steering Committee, 1991).

### Results

### *Patients in the trial*

Between February 1985 and April 1989, 491 patients were admitted to the trial from 21 centres in the United Kingdom. Of these, 33 were ineligible (Paper 1), leaving 458 (157 ECMV3, 152 ECMV6, 149 EI6) for analysis.

The main symptoms, overall condition and level of physical activity of the patients as assessed on admission by the clinicians are shown in Table II. Most (84%) of the patients had cough (moderate or severe in 38%), and 31% had haemoptysis, 47% chest pain, 51% anorexia, and 8% dysphagia. The overall condition, level of physical activity, and degree of breathlessness were normal or nearly normal (grade 0 or 1) in 62%, 63%, and 52%, respectively. The distributions of all these variables were similar in the three treatment groups.

### *Clinicians' and patients' compliance in completing forms and diary cards*

The compliance by clinicians in providing quality of life data at the time of clinic attendances by patients (Table III) was high, 90% of the expected data being provided. The centre that provided no data entered only a single patient. All other centres provided more than 50% of data and 11 provided 90% or more. In marked contrast, only 47% of the expected data from the patient diary cards was received, a third of the patients providing no data at all, and only a third providing more than 75% of the expected data. There was considerable

**Table II** Characteristics of the patients on admission as recorded by the clinicians

| | *ECMV3* | | *ECMV6* | | *EI6* | | *Total* | |
|---|---|---|---|---|---|---|---|---|
| *Characteristic* | *No.* | *(%)* | *No.* | *(%)* | *No.* | *(%)* | *No.* | *(%)* |
| *Cough* | | | | | | | | |
| None | 17 | (11) | 23 | (15) | 32 | (22) | 72 | (16) |
| Mild | 73 | (46) | 69 | (46) | 68 | (46) | 210 | (46) |
| Moderate | 64 | (41) | 52 | (35) | 41 | (28) | 157 | (35) |
| Severe | 3 | (2) | 6 | (4) | 6 | (4) | 15 | (3) |
| Not known | 0 | | 2 | | 2 | | 4 | |
| *Haemoptysis* | | | | | | | | |
| None | 97 | (63) | 106 | (71) | 110 | (75) | 313 | (69) |
| Mild | 52 | (34) | 36 | (24) | 28 | (19) | 116 | (26) |
| Moderate | 5 | (3) | 8 | (5) | 9 | (6) | 22 | (5) |
| Severe | 0 | (0) | 0 | (0) | 0 | (0) | 0 | (0) |
| Not known | 3 | | 2 | | 2 | | 7 | |
| *Chest pain* | | | | | | | | |
| None | 79 | (51) | 86 | (58) | 75 | (51) | 240 | (53) |
| Mild | 43 | (28) | 32 | (21) | 44 | (30) | 119 | (26) |
| Moderate | 30 | (19) | 26 | (17) | 22 | (15) | 78 | (17) |
| Severe | 4 | (3) | 5 | (3) | 5 | (3) | 14 | (3) |
| Not known | 1 | | 3 | | 3 | | 7 | |
| *Anorexia* | | | | | | | | |
| None | 74 | (48) | 72 | (49) | 74 | (51) | 220 | (49) |
| Mild | 54 | (35) | 44 | (30) | 36 | (25) | 134 | (30) |
| Moderate | 24 | (15) | 24 | (16) | 31 | (21) | 79 | (18) |
| Severe | 3 | (2) | 7 | (5) | 5 | (3) | 15 | (3) |
| Not known | 2 | | 5 | | 3 | | 10 | |
| *Dysphagia* | | | | | | | | |
| None | 143 | (95) | 131 | (90) | 133 | (92) | 407 | (92) |
| Mild | 5 | (3) | 8 | (5) | 7 | (5) | 20 | (5) |
| Moderate | 3 | (2) | 5 | (3) | 1 | (1) | 9 | (2) |
| Severe | 0 | (0) | 2 | (1) | 3 | (2) | 5 | (1) |
| Not known | 6 | | 6 | | 5 | | 17 | |
| *Overall condition*[a] | | | | | | | | |
| 0 | 15 | (10) | 18 | (12) | 20 | (14) | 53 | (12) |
| 1 | 75 | (49) | 76 | (51) | 72 | (50) | 223 | (50) |
| 2 | 55 | (36) | 43 | (29) | 38 | (26) | 136 | (30) |
| 3 | 8 | (5) | 9 | (6) | 14 | (10) | 31 | (7) |
| 4 | 1 | (1) | 2 | (1) | 1 | (1) | 4 | (1) |
| Not known | 3 | | 4 | | 4 | | 11 | |
| *Level of physical activity*[a] | | | | | | | | |
| 0 | 28 | (18) | 27 | (19) | 29 | (20) | 84 | (19) |
| 1 | 68 | (44) | 63 | (43) | 65 | (45) | 196 | (44) |
| 2 | 46 | (30) | 38 | (26) | 32 | (22) | 116 | (26) |
| 3 | 10 | (7) | 15 | (10) | 17 | (12) | 42 | (10) |
| 4 | 1 | (1) | 2 | (1) | 1 | (1) | 4 | (1) |
| Not known | 4 | | 7 | | 5 | | 16 | |
| *Degree of breathlessness*[a] | | | | | | | | |
| 0 | 27 | (18) | 22 | (15) | 35 | (24) | 84 | (19) |
| 1 | 45 | (30) | 54 | (37) | 46 | (31) | 145 | (33) |
| 2 | 37 | (24) | 36 | (24) | 35 | (24) | 108 | (24) |
| 3 | 33 | (22) | 27 | (18) | 20 | (14) | 80 | (18) |
| 4 | 10 | (7) | 8 | (50) | 11 | (7) | 29 | (7) |
| Not known | 5 | | 5 | | 2 | | 12 | |

[a]Definitions given in Table I.

variation between the 21 centres in providing patient diary card data. All centres provided some data but three provided only 25% or less. At the other end of the range, the three most compliant centres provided 77, 78 and 91% of data.

Compliance in the provision of patient diary card data (Table IV) was higher (52%) during the first 9 weeks, the period of the first three courses of chemotherapy, than subsequently (43%). It was unaffected by the age of patients; male patients provided 49% of their expected data and female 42%. Patients with limited disease on admission provided less of their data (43%) than those with extensive disease (53%). That this was due to the change in supervision associated with receiving radiotherapy after three courses of chemotherapy in patients with limited disease is suggested by the observation that the least compliant group with respect to extent of disease were patients with limited disease during weeks 10 to 21 when they provided 38% of data.

Performance status had a major effect on compliance which ranged from 56% in patients with a good status (grade 0) on admission down to only 19% in those with a poor status (grade 4). Somewhat unexpectedly, compliance was worse (41%) with the three-course regimen than with the two six-course regimens (51%, 49%). The reason for this may in part be that patients in the ECMV3 group were discharged from specialist care earlier than those in the ECMV6 and EI6 groups: the least compliant patients with respect to regimen were the ECMV3 patients during weeks 10 to 21, after the end of their three courses of chemotherapy, when they provided 36% of data.

**Table III** Compliance by centres and patients in providing quality of life data[a]

| Percentage of data provided | *Data from clinicians' reports* Centres No. | (%) | *Patient diary card data* Patients No. | (%) | Centres No. | (%) |
|---|---|---|---|---|---|---|
| None | 1 | (5) | 128 | (31) | 0 | (0) |
| 1–25 | 0 | (0) | 32 | (8) | 3 | (14) |
| 26–50 | 0 | (0) | 62 | (15) | 8 | (38) |
| 51–75 | 3 | (14) | 60 | (15) | 7 | (33) |
| 76–100 | 17 | (81) | 127 | (31) | 3 | (14) |
| Overall percentage of data received | | (90) | | (47) | | (47) |

[a]Based on the 409 eligible patients who survived at least 4 weeks from randomisation.

**Table IV** Percentage of diary card data provided according to patients' characteristics on admission

| *Characteristic* | *Number of patients* | *Percentage of data provided during weeks* 0–9 | 10–21 | 0–21 |
|---|---|---|---|---|
| *Age* | | | | |
| -44 | 17 | 50 | 43 | 46 |
| 45–54 | 64 | 51 | 42 | 46 |
| 55–64 | 189 | 55 | 45 | 49 |
| 65–75 | 139 | 48 | 40 | 44 |
| *Sex* | | | | |
| Male | 270 | 54 | 45 | 49 |
| Female | 139 | 47 | 38 | 42 |
| *Extent of disease* | | | | |
| Limited | 241 | 49 | 38 | 43 |
| Extensive | 168 | 56 | 50 | 53 |
| *Performance status* | | | | |
| 0 | 83 | 62 | 52 | 56 |
| 1 | 188 | 55 | 44 | 49 |
| 2 | 93 | 45 | 40 | 42 |
| 3 | 30 | 44 | 31 | 37 |
| 4 | 2 | 30 | 2 | 19 |
| Not known | 13 | 6 | 4 | 5 |
| *Regimen* | | | | |
| ECMV3 | 144 | 47 | 36 | 41 |
| ECMV6 | 133 | 56 | 47 | 51 |
| EI6 | 132 | 52 | 45 | 49 |
| All patients | 409 | 52 | 43 | 47 |

*Adverse reactions to treatment*

The main adverse reactions, other than alopecia, that were reported by the clinicans as being moderate or severe during chemotherapy are shown in Table V. The findings were, in general, very similar in the three treatment groups: 59% of the ECMV3, 67% of the ECMV6, and 63% of the EI6 patients having one or more moderate or severe reactions. The commonest were anorexia, myelosuppression (mainly anaemia and leucopenia), dysphagia, and vomiting. Anorexia was reported in a higher proportion of patients in the ECMV6 group (36%) than in the other two groups (ECMV3 29%, EI6 24%). However, unlike the other symptoms of adverse reactions, anorexia was reported in substantial proportions of patients pretreatment (Table II). When patients with moderate or severe anorexia pretreatment are excluded, the proportions of patients with this symptom reported as an adverse reaction to treatment were similar in the three groups, namely, 22% of the ECMV3, 23% of the ECMV6, and 20% of the EI6 patients.

*Clinicians' assessments of palliation of symptoms*

Palliation of the main symptoms (Table VI) was achieved in high proportions of patients, ranging in the ECMV3 group from 63% for dysphagia to 89% for haemoptysis, in the ECMV6 group from 74% for cough to 91% for haemoptysis, and in the EI6 group from 73% for dysphagia to 86% for haemoptysis. In the majority of patients palliation of a symptom involved disappearance of that symptom. The proportions of patients in whom palliation was achieved and in whom symptoms disappeared were similar in the three treatment groups.

The median number of days in palliation (right-hand part of Table VI) ranged in the ECMV3 group from 94 for cough to 164 for haemoptysis, in the ECMV6 group from 109 for dysphagia to 162 for haemoptysis, and in the EI6 group from 126 for cough to 189 for haemoptysis. For all five of the symptoms the median duration of palliation was 63% or more of survival during the first year. The findings in the three treatment groups were very similar.

*Clinicians' assessments of overall condition, level of physical activity, and breathlessness*

The grades of overall condition, physical activity, and breathlessness as assessed by the clinicians are defined in Table I. Among patients with grade 1 or worse on admission (Table

**Table V** Main adverse reactions other than alopecia reported by the clinicians to be moderate or severe; based on the 443 patients who started their allocated treatment

| *Reaction* | *ECMV3* No. | (%) | *ECMV6* No. | (%) | *EI6* No. | (%) |
|---|---|---|---|---|---|---|
| Anorexia | 44 | (29) | 54 | (36) | 34 | (24) |
| Nausea (without vomiting) | 15 | (10) | 17 | (11) | 13 | (9) |
| Vomiting | 20 | (13) | 25 | (17) | 14 | (10) |
| Dysphagia | 14 | (9) | 25 | (17) | 23 | (16) |
| Sore mouth | 22 | (14) | 26 | (17) | 11 | (8) |
| Diarrhoea | 12 | (8) | 9 | (6) | 9 | (6) |
| Cystitis | 3 | (2) | 5 | (3) | 3 | (2) |
| Paraesthesia | 13 | (9) | 15 | (10) | 8 | (6) |
| Haematological (WHO grade 2 or worse) | | | | | | |
| anaemia ($Hb \leqslant 9.4\ g\ dl^{-1}$) | 20 | (13) | 32 | (21) | 27 | (19) |
| leucopenia ($WBC \leqslant 2.9 \times 10^3\ mm^{-3}$) | 15 | (10) | 31 | (21) | 24 | (17) |
| thrombocytopenia (platelets $\leqslant 74 \times 10^3\ mm^{-3}$) | 5 | (3) | 8 | (5) | 1 | (1) |
| Total patients with any of the above reactions | 89 | (59) | 100 | (67) | 90 | (63) |
| Total patients | 152 | (100) | 149 | (100) | 142 | (100) |

VII), the proportions who improved tended to be higher in the E16 group although the differences were small. Thus, for overall condition, the proportions with improvement were 49% in the ECMV3 group and 40% in the ECMV6 group compared with 55% in the EI6 group. The corresponding figures for level of physical activity were 43%, 48%, and 59% and for degree of breathlessness, 58%, 56% and 71%. There was no evidence of any consistent difference between the treatment groups in the duration of improvement.

*Clinicians' assessments of anxiety and depression*

The proportions of patients with anxiety and with depression on admission and at the three subsequent assessments are shown in Table VIII. To avoid possible distortion of the results by early attrition, this analysis is limited to the patients with all the relevant data available. On admission, 17% of the ECMV3, 16% of the ECMV6 and 9% of the EI6 patients were reported by their clinicians to be moderately anxious (grade 2), and a further 8%, 3% and 4%, respectively, severely so (grade 3). However, at the following three assessments these levels of anxiety had fallen substantially. The proportions of patients reported to be moderately or severely depressed were lower and remained so during this period. There was no evidence of a consistent difference between the treatment groups with respect to either anxiety or depression. Both anxiety and depression were somewhat commoner and more severe in females than in males pretreatment, but the proportions with improvement were similar (details not shown).

**Table VI** Palliation of main symptoms as assessed by clinicians

| Symptom | Regimen | No. of Patients with symptom pretreatment | Patients with palliation No. | (%) | Patients in whom symptom disappeared No. | (%) | Patients with palliation: Time (days) in palliation Median | $Q^a$ | Proportion of survival in palliation in the first year Median % | $Q^a$ |
|---|---|---|---|---|---|---|---|---|---|---|
| Cough | ECMV3 | 140 | 112 | (80) | 91 | (65) | 94 | 51-198 | (66) | 43-90 |
| | ECMV6 | 127 | 94 | (74) | 83 | (65) | 131 | 66-198 | (72) | 39-92 |
| | EI6 | 115 | 95 | (83) | 87 | (76) | 126 | 69-196 | (63) | 44-84 |
| Haemoptysis | ECMV3 | 57 | 51 | (89) | 51 | (89) | 164 | 74-221 | (90) | 79-94 |
| | ECMV6 | 44 | 40 | (91) | 39 | (89) | 162 | 74-226 | (89) | 64-95 |
| | EI6 | 37 | 32 | (86) | 32 | (86) | 189 | 140-234 | (92) | 81-96 |
| Chest pain | ECMV3 | 77 | 66 | (86) | 64 | (83) | 115 | 59-185 | (75) | 51-87 |
| | ECMV6 | 63 | 54 | (87) | 51 | (82) | 125 | 68-197 | (78) | 50-91 |
| | EI6 | 71 | 58 | (82) | 54 | (76) | 169 | 94-225 | (82) | 58-94 |
| Anorexia | ECMV3 | 81 | 63 | (78) | 58 | (72) | 129 | 64-189 | (70) | 50-83 |
| | ECMV6 | 75 | 57 | (76) | 54 | (72) | 120 | 79-168 | (70) | 50-85 |
| | EI6 | 72 | 57 | (79) | 54 | (75) | 175 | 70-220 | (77) | 50-91 |
| Dysphagia | ECMV3 | 8 | 5 | (63) | 5 | (63) | 141 | 37-233 | (88) | 67-90 |
| | ECMV6 | 15 | 12 | (80) | 11 | (73) | 109 | 66-191 | (71) | 52-83 |
| | EI6 | 11 | 8 | (73) | 8 | (73) | 149 | 10-218 | (86) | 50-94 |

[a]Q = interquartile range.

**Table VII** Overall condition, performance status, and degree of breathlessness as assessed by clinicians

| Assessment | Regimen | Patients with grade 1 or worse on admission | Patients with improvement No. | (%) | Time (days) improved Median | $Q^a$ | Proportion of survival time improved Median % | $Q^a$ |
|---|---|---|---|---|---|---|---|---|
| Overall condition | ECMV3 | 139 | 68 | (49) | 89 | 42-147 | (52) | 25-72 |
| | ECMV6 | 129 | 52 | (40) | 79 | 40-121 | (41) | 22-62 |
| | EC6 | 125 | 69 | (55) | 81 | 42-159 | (51) | 18-76 |
| Performance status (WHO) | ECMV3 | 125 | 54 | (43) | 98 | 59-160 | (52) | 30-76 |
| | ECMV6 | 117 | 56 | (48) | 80 | 28-142 | (48) | 19-74 |
| | EI6 | 115 | 68 | (59) | 107 | 53-173 | (50) | 30-81 |
| Degree of breathlessness | ECMV3 | 125 | 72 | (58) | 113 | 48-188 | (68) | 37-87 |
| | ECMV6 | 124 | 69 | (56) | 84 | 56-157 | (50) | 31-80 |
| | EI6 | 112 | 79 | (71) | 107 | 53-163 | (50) | 32-76 |

[a]Q = interquartile range.

**Table VIII** Clinicians' assessments of anxietey and depression: percentages of patients in each grade; based on patients with data available at all four assessments

| | Number of patients | Pretreatment Grade 0 | 1 | 2 | 3 | Follow-up 1 0 | 1 | 2 | 3 | Follow-up 2 0 | 1 | 2 | 3 | Follow-up 3 0 | 1 | 2 | 3 |
|---|---|---|---|---|---|---|---|---|---|---|---|---|---|---|---|---|---|
| *Anxiety* | | | | | | | | | | | | | | | | | |
| ECMV3 | 96 | 36 | 39 | 17 | 8 | 56 | 33 | 8 | 2 | 58 | 31 | 10 | 0 | 54 | 39 | 7 | 0 |
| ECMV6 | 87 | 45 | 36 | 16 | 3 | 59 | 30 | 9 | 2 | 68 | 26 | 6 | 0 | 60 | 30 | 9 | 1 |
| EI6 | 96 | 56 | 30 | 9 | 4 | 59 | 31 | 8 | 1 | 57 | 32 | 8 | 2 | 68 | 25 | 7 | 0 |
| *Depression* | | | | | | | | | | | | | | | | | |
| ECMV3 | 96 | 66 | 23 | 10 | 1 | 70 | 22 | 7 | 1 | 70 | 23 | 6 | 1 | 69 | 22 | 8 | 1 |
| ECMV6 | 85 | 67 | 32 | 1 | 0 | 69 | 26 | 4 | 1 | 76 | 20 | 4 | 0 | 64 | 33 | 2 | 1 |
| EI6 | 96 | 76 | 18 | 5 | 1 | 69 | 23 | 8 | 0 | 71 | 24 | 4 | 1 | 75 | 17 | 8 | 0 |

*Patients' assessments using the diary cards*

The quality of life during the first 9 weeks from the date of randomisation (the period of the first three courses of chemotherapy), as recorded by patients on their diary cards, is expressed in Table IX as percentages of patient-days for each category. The results for the three treatment groups were very similar for nausea, vomiting, and activity. There was some indication that the results for dysphagia and for mood were a little better for the EI6 regimen than for the ECMV regimens but the differences were small. The results for overall condition were better for the EI6 regimen, the percentages for the two better categories (0 and 1) combined being 59% compared with 43% for the two ECMV regimens.

**Table IX** Patients' assessment of quality of life during the first 9 weeks from the date of randomisation; based on all available data

| | | *Percentage of patient-days Category recorded on diary card*[a] | | | | |
|---|---|---|---|---|---|---|
| *Assessment* | *Regimen* | *0* | *1* | *2* | *3* | *4* |
| Nausea | ECMV3 | 78 | 15 | 5 | 2 | – |
| | ECMV6 | 79 | 15 | 4 | 2 | – |
| | EI6 | 76 | 17 | 6 | 2 | – |
| Vomiting | ECMV3 | 90 | 5 | 3 | 2 | – |
| | ECMV6 | 92 | 4 | 3 | 2 | – |
| | EI6 | 90 | 5 | 3 | 3 | – |
| Dysphagia | ECMV3 | 86 | 8 | 4 | 2 | 0 |
| | ECMV6 | 85 | 11 | 2 | 2 | 0 |
| | EI6 | 93 | 4 | 2 | 0 | 0 |
| Activity | ECMV3 | 15 | 14 | 42 | 25 | 4 |
| | ECMV6 | 16 | 13 | 40 | 28 | 3 |
| | EI6 | 17 | 13 | 39 | 26 | 4 |
| Mood | ECMV3 | 7 | 30 | 50 | 11 | 2 |
| | ECMV6 | 7 | 30 | 52 | 11 | 1 |
| | EI6 | 10 | 36 | 45 | 9 | 1 |
| Overall condition | ECMV3 | 11 | 32 | 46 | 10 | 2 |
| | ECMV6 | 9 | 34 | 46 | 10 | 1 |
| | EI6 | 15 | 43 | 34 | 8 | 1 |

[a]The categories are listed in Table I.

The diary card data are displayed graphically day by day in Figures 1, 2 and 3.

Figure 1 presents the same data as are shown in Table IX, namely data for all patients during the first three courses of chemotherapy. For all six variables the relationship to the three courses of chemotherapy is clear, a deterioration being seen during the days on which chemotherapy was given, although this was less marked for mood than for the others. The patterns for nausea, vomiting, activity, and mood were very similar in the three treatment groups. There was somewhat more dysphagia in the two ECMV groups than in the EI6 group. Nausea tended to persist for longer than vomiting between courses of chemotherapy. Overall condition was substantially better for the EI6 group between courses of chemotherapy, suggesting that patients in this group recovered more rapidly from the adverse effects of each course.

Figures 2 and 3 display the data over the whole treatment period in the subgroups of patients with limited disease (Figure 2) and extensive disease (Figure 3) on admission. The effects of stopping chemotherapy after three courses in the ECMV3 group are evident, the overall levels of all six variables remaining essentially steady after the third course in this group compared with the other two. A comparison of the two figures indicates a considerable, but short-lived, in-

**Figure 1** Percentages of patients with grade 2 or worse nausea, vomiting, dysphagia, mood, and overall condition, and grade 3 or worse level of physical activity: EMCV3 (———), EMCV6 (- - -), EI6 (– – –). Data from all patients during the first three courses of chemotherapy (ct1, ct2, and ct3).

**Figure 2** As for Figure 1. Data from patients with limited disease on admission during the whole treatment period ('rt start' and 'rt end' indicate the period of thoracic radiotherapy).

crease in dysphagia during thoracic radiotherapy in patients with limited disease. Both figures show that with the fourth, fifth, and sixth courses of chemotherapy, recovery after the course was more rapid in the EI6 than the ECMV6 group, reinforcing the conclusion from Figure 1.

All these figures show high proportions of patients in all three groups with a low level of activity for the first day. This is in marked contrast to the clinicians' assessments pretreatment (Table II). This discrepancy almost certainly arose because patients reported their activity level at the end of the first day of intravenous chemotherapy while clinicians made their assessment before it was started.

### Discussion

This trial emphasises both the importance and the feasibility of studying toxicity, the palliation of symptoms and other aspects of quality of life in randomised comparisons between treatment regimens in the management of lung cancer. It is particularly important to study these endpoints in patients with poor prognosis. Despite the inherent problems of attrition and of obtaining data in this group of patients, provided there are no treatment-related imbalances in the data, valid comparisons can be made between treatment groups. Comparisons of such endpoints need to be made in randomised trials because they may have an important bearing on treatment policies, and results can be counter-intuitive (Slevin, 1992). For example, in a randomised trial in which all patients received the same palliative drug combination for small-cell lung cancer, the regimen was allocated at random to be given either at conventional 3-week intervals or only as required to control progressive disease and relieve symptoms (Earl *et al.*, 1991). It was expected that patients in the latter group might require less total chemotherapy and therefore enjoy better quality of life. In the event, less chemotherapy was indeed given to this group but palliation of symptoms was substantially less effective and quality of life was worse.

Quality of life analyses can be compromised by poor compliance in providing data. For example, Ganz *et al.* (1988) reported such a low level of compliance by patients in completing the Functional Living Index – Cancer (FLIC) questionnaire at 4-weekly intervals that they were unable to use FLIC data in their comparison of regimen.

In the present trial, clinicians recorded their assessments of the presence and severity (mild, moderate or severe) of patients' symptoms and of physical activity, mood, and overall condition at clinic attendances for chemotherapy and follow-up. Their level of compliance in recording these assessments was high, 90% of the expected data being provided.

With all three regimens – etoposide, cyclophosphamide, methotrexate, and vincristine for three courses (ECMV3) or six courses (ECMV6), or etoposide and ifosfamide for six courses (EI6) – about two thirds of the patients were reported by their clinicians to have experienced moderate or

**Figure 3** As for Figure 1. Data from patients with extensive disease on admission during the whole treatment period.

severe adverse effects of treatment, the commonest of which were anorexia, myelosuppression, dysphagia, and vomiting. The proportions of patients with reactions were very similar in the three treatment groups, but patients in the 6-course groups were potentially exposed to reactions on twice as many occasions as those in the 3-course group. In this respect, there was therefore an advantage to the 3-course regimen.

As reported by the clinicians, all three regimens were highly effective in palliating the symptoms of the disease. Cough, haemoptysis, chest pain, anorexia, and dysphagia were each palliated in 63% or more of the affected patients, the symptom disappearing at least for a time in 57% or more. Moreover, the median duration of palliation was 63% or more of survival for all the above-mentioned symptoms. The proportions of patients with improved overall condition, level of physical activity, and breathlessness were somewhat higher in the EI6 group than in the two ECMV groups, but the differences were small.

The clinicians' assessments of anxiety and depression consisted of recording whether each was absent, mild, moderate, or severe. In all three treatment groups, about a third of the patients were reported as having mild anxiety on admission, 14% moderate anxiety, and 5% severe anxiety, but these proportions were substantially reduced over the next three clinic attendances, suggesting that anxiety is considerably alleviated by palliative treatment. Some 24% of patients were reported to be mildly depressed on admission and 5% moderately depressed, but less than 1% severely depressed. In contrast to the findings for anxiety, these proportions remained similar at subsequent assessments. They should alert clinicians to the possibility that a small proportion of patients might benefit from a more detailed psychological assessment with a view to considering specific antidepressant treatment. As reported by clinicians in the present trial, both anxiety and depression were somewhat commoner and more severe in female patients, but the proportions with improvement were similar. The method of assessing anxiety and depression was a relatively insensitive one. In current trials, therefore, the MRC Lung Cancer Working Party are using the HAD (Hospital Anxiety and Depression) scale which has been shown to be reliable and valid (Zigmond & Snaith, 1983). We are also monitoring the use of psychotropic drugs. Use of HAD scale data will also enable us to make better comparisons between female and male patients.

An important feature of the trial was the use of the daily diary card (Fayers *et al.*, 1991). This assessment by the patients of nausea, vomiting, difficulty in swallowing, physical activity, mood, and overall condition was completed every evening after their last meal for the first 21 weeks in the trial. It has been shown to be reliable and to be sensitive to day-to-day changes in major symptoms (Fayers *et al.*, 1991; MRC Lung Cancer Working Party, 1991; 1992). Nevertheless, only 47% of the requested data was provided. The factor that most influenced compliance was performance status on admission, patients with a better status complying better. There was substantial variation in compliance between centres. This suggests that hospital and clinic staff could have an important influence on improving compliance. The trial protocol emphasised the need to explain to patients the importance of completing their cards in arriving at a thorough assessment of their illness and its treatment. No formal attempt was made to check on this, but informal discussions with centres suggested that compliance is best when clinicians or nurses encourage patients to complete their cards and then discuss with them what they have recorded.

Other trials have also reported difficulty in collecting quality of life data from patients with a poor performance status and progressing disease (Ganz *et al.*, 1988; 1989; Geddes *et al.*, 1990; MRC Lung Cancer Working Party, 1992). It seems likely that this will remain a limitation of methods dependent on collecting data direct from very ill patients themselves. Patients allocated to the six-course regimens complied somewhat better then those assigned to the three-course regimen. This was an unexpected finding and probably occurred because patients in the three-course group were likely to have been discharged to non-specialist care sooner than those in the six-course groups. This emphasises the need to explain to patients the value of daily assessments and to encourage them to complete them.

The trial exemplifies the sensitivity of the daily diary card in detecting day-to-day variation. The findings for the three regimens were very similar for nausea, vomiting, and level of physical activity. Recovery from vomiting was rapid after a course of chemotherapy in all three groups, confirming previous findings during chemotherapy with the ECMV regimen (Fayers *et al.*, 1991), but nausea and reduced physical activity tended to persist for longer. It will therefore be important to see whether they limit the use of chemotherapy schedules in which drugs are given in reduced dosage once a week (for example Miles *et al.*, 1991), or in which dose intensity is increased by giving the drugs in full dosage once every 2 weeks together with haemopoietic growth factor, a policy which the MRC Lung Cancer Working Party is currently studying.

The EI6 regimen caused somewhat less dysphagia than the ECMV regimens, and between courses of chemotherapy patients reported themselves to be in better overall condition. Nevertheless, these advantages need to be weighed against the inconvenience of the 24 h infusions required, compared with the 30 min infusions of the ECMV regimens.

Clearly it is important to find and develop the use of quality of life instruments that are acceptable, relevant and applicable. Apart from the daily diary card, no patient questionnaires were used in the present trial, but in its current trials the MRC Lung Cancer Working Party are using the Rotterdam Symptom Checklist (de Haes *et al.*, 1990) and the HAD scale with high levels of compliance by patients.

One limitation of the design of the present trial is that it did not permit a reliable comparison to be made between clinicians' and patients' assessments of quality of life because assessments were made daily by patients but intermittently by clinicians; also, the questions asked of each were not the same. Nevertheless, this is an important methodological issue (Slevin *et al.*, 1988) and is being addressed in current MRC Lung Cancer Working Party trials in which some of the questions on the Rotterdam Symptom Checklist are duplicated on the reports completed by clinicians.

In conclusion, although not easy, it is important to study palliative and other quality of life endpoints in trials of chemotherapy in the treatment of small-cell lung cancer. This is especially necessary when, as in the present trial, a principal aim is to improve the quality of survival. The findings presented in this paper and in Paper 1 show that there was no major clinical gain from continuing chemotherapy beyond three courses or from using the ifosfamide regimen. Nevertheless, they do not exclude the possibility of a minor survival advantage with the six-course regimens (Paper 1). Even small chances of longer survival may be important to patients but the six-course regimens involve some 5 or 6 months of treatment in a patient population with a median survival of only about 9 months. Some patients might therefore prefer a shorter treatment period. All three regimens produced high and similar levels of palliation of the main chest symptoms, but there was a suggestion of a minor advantage to the EI6 regimen with respect to overall condition, physical activity, breathlessness and dysphagia.

The MRC Lung Cancer Working Party are currently studying palliative regimens of intravenous etoposide and vincristine and of orally administered etoposide alone with the aim of achieving high levels of palliation and low levels of toxicity in a large programme of trials of palliative treatments for lung cancer.

The following consultants and their colleagues entered 20 or more patients into the trial: Brighton: J.P.R. Hartley, N.J. Hodson, C.W. Turton; Bristol: V.L. Barley, J.A. Bullimore, R.J. White; Cambridge: N.M. Bleehen, M.V. Williams; Cork: C.P. Bredin; Kettering: A.R. Davidson, T.J. Williams; Leeds: D.V. Ash, H.J. Close, C.A. Joslin, M.F. Muers, J. Stone; Mount Vernon: R.F. Ashford, S. Dische, E.P. Dunphy, D.C. Fermont, E. Grosch, E.J. Maher, M.I. Saunders; Oxford: R.J. Adam, C.J. Alcock, M.K. Benson, J.M. Hopkin, D.J. Lane; York: A.M. Hunter.

The remaining patients were entered by the following consultants and their colleagues: Carluke: J.C.J.L. Bath; Chesterfield: J.W. Hadfield; Inverness: W.D. Murray; Ipswich: C.R. Wiltshire; Middlesex; R. Berry, A.M. Jelliffe, A.R. Makepeace, M.F. Spittle; Milton Keynes: S. Fisher; Northampton: G.C. Ferguson; Plymouth: J.M. Brindle, A.F. Broad, C.R. McGavin; Sheffield: J.J. Bolger, A.E. Champion, K. Dunn, I.H. Manifold; Stoke Mandeville: S.J. Williams; Swindon: J.A. Waddell; Wolverhampton: D.J. Fairlamb.

The reference histopathologist was P.G.I. Stovin.

Local coordinators were: A. Anderson, R. Collins, L. Crossley, C. des Rochers, A. Fenwick, S. Garner, L. Grant, C. Hutchinson, V. Marmur, A. Pickett, D. Robinson, C. Schuerman, K. Weiner and T. Young.

The MRC Trials Office data managers were: Elizabeth Brodnicki, Grazyna Lallemand and Sheila Thornton.

### References

BERGMAN, B. (1992). Psychosocial issues in the treatment of patients with lung cancer. *Lung Cancer*, **8**, 1–20.

BROCK, N. & POHL, J. (1983). The development of mesna for regional detoxification. *Cancer Treat. Revs.*, **10** (Supplement A), 33–43.

COMPACT STEERING COMMITTEE (1991). Improving the quality of clinical trials in cancer. *Br. J. Cancer*, **63**, 412–415.

CULLEN, M., MORGAN, D., GREGORY, W., ROBINSON, M., COX, D., MCGIVERN, D., WARD, M., RICHARDS, M., STABLEFORTH, D., MACFARLANE, A., STIRLAND, J., WOODROFFE, C., MACFARLANE, J., FLETCHER, J., DAVIES, D. AND THE MIDLANDS SMALL CELL LUNG CANCER GROUP (1986). Maintenance chemotherapy for anaplastic small cell carcinoma of the bronchus: a randomised controlled trial. *Cancer Chemother. Pharmacol.*, **17**, 157–160.

DE HAES, J.C.J., KNIPPENBERG, F.C.E. & NEIJT, J.P. (1990). Measuring psychological and physical distress in cancer patients: structure and application of the Rotterdam Symptom Checklist. *Br. J. Cancer*, **2**, 1034–1038.

EARL, H.M., RUDD, R.M., SPIRO, S.G., ASH, C.M., JAMES, L.E., LAW, C.S., TOBIAS, J.S., HARPER, P.G., GEDDES, D.M., ERAUT, D., PARTRIDGE, M.R. & SOUHAMI, R.L. (1991). A randomised trial of planned versus as required chemotherapy in small cell lung cancer: a Cancer Research Campaign trial. *Br. J. Cancer*, **64**, 566–572.

FAYERS, P.M., BLEEHEN, N.M., GIRLING, D.J. & STEPHENS, R.J. (1991). Assessment of quality of life in small-cell lung cancer using a daily diary card developed by the Medical Research Council Lung Cancer Working Party. *Br. J. Cancer*, **64**, 299–306.

FAYERS, P.M. (1992). Quality-of-life assessment in small cell lung cancer. *PharmacoEconomics*, **2**, 181–188.

GANZ, P.A., FIGLIN, R.A., HASKELL, C.M. LA SOTO, N. & SIAU, J. FOR THE UCLA SOLID TUMOR STUDY GROUP (1989). Supportive care versus supportive care and combination chemotherapy in metastatic non-small cell lung cancer: does chemotherapy make a difference? *Cancer*, **63**, 1271–1278.

GANZ, P.A., HASKELL, C.M., FIGLIN, R.A., LA SOTO, N. & SIAU, J. FOR THE UCLA SOLID TUMOR STUDY GROUP (1988). Estimating the quality of life in a clinical trial of patients with metastatic lung cancer using the Karnofsky performance status and the functional living index-cancer. *Cancer*, **61**, 849-856.

GEDDES, D.M., DONES, L., HILL, E., LAW, K., HARPER, P.G., SPIRO, S.G., TOBIAS, J.S. & SOUHAMI, R.L. (1990). Quality of life during chemotherapy for small cell lung cancer: assessment and use of a daily diary card in a randomised trial. *Eur. J. Cancer*, **26**, 484-492.

GIACCONE, G., DALESIO, O., MCVIE, G.J., KIRKPATRICK, A., POSTMUS, P.E., BURGHOUTS, J.T.M., BAKKER, W., KOOLEN, M.G.J., VENDRIK, C.P.J., ROOZENDAAL, K.J., PLANTING, A.S.T., VAN ZANDWIJK, N., TEN VELDE, G.J.M. & SPLINGER, A.W. FOR THE EUROPEAN ORGANIZATION FOR RESEARCH AND TREATMENT OF CANCER LUNG CANCER COOPERATIVE GROUP. Maintenance chemotherapy in small cell lung cancer: long-term results of a randomized trials. *J. Clin. Oncol.*, **11**, 1230-1240.

KARNOFSKY, D. & BURCHENAL, J.H. (1949). Clinical Evaluation of Chemotherapeutic Agents in Cancer. In: McLeod, C.M. (ed). *Evaluation of Chemotherapeutic Agents*. Columbia University Press: New York.

MEDICAL RESEARCH COUNCIL LUNG CANCER WORKING PARTY (1989). Controlled trial of twelve versus six courses of chemotherapy in the treatment of small-cell lung cancer. *Br. J. Cancer*, **59**, 584-590.

MEDICAL RESEARCH COUNCIL LUNG CANCER WORKING PARTY (1991). Inoperable non-small-cell lung cancer (NSCLC): a Medical Research Council randomised trial of palliative radiotherapy with two fractions or ten fractions. *Br. J. Cancer*, **63**, 265-270.

MEDICAL RESEARCH COUNCIL LUNG CANCER WORKING PARTY (1992). A Medical Research Council (MRC) randomised trial of palliative radiotherapy with two fractions or a single fraction in patients with inoperable non-small-cell lung cancer (NSCLC) and poor performance status. *Br. J. Cancer*, **65**, 934-941.

MEDICAL RESEARCH COUNCIL LUNG CANCER WORKING PARTY (1993*a*). A randomised trial of 3 or 6 courses of etoposide cyclophosphamide methotrexate and vincristine or 6 courses of etoposide and ifosfamide in small-cell lung cancer (SCLC) I: survival and prognostic factors. *Br. J. Cancer*, **68**, 1150-1156.

MILES, D.W., EARL, H.M., SOUHAMI, R.L., HARPER, P.G., RUDD, R., ASH, C.M., JAMES, L., TRASK, C.W.L., TOBIAS, J.S. & SPIRO, S.G. (1991). Intensive weekly chemotherapy for good-prognosis patients with small-cell lung cancer. *J. Clin. Oncol.*, **9**, 280-285.

SLEVIN, M.L., PLANT, H., LYNCH, D., DRINKWATER, J. & GREGORY, W.M. (1988). Who should measure quality of life, the doctor or the patient? *Br. J. Cancer*, **57**, 109-112.

SLEVIN, M.L. (1992). Current issues in cancer - quality of life: philosophical question or clinical reality? *Br. Med. J.*, **305**, 466-469.

SPIRO, S.G., SOUHAMI, R.L., GEDDES, D.M., ASH, C.M., QUINN, H., HARPER, P.G., TOBIAS, J.S. PARTRIDGE, M. & ERAUT, D. (1989). Duration of chemotherapy in small cell lung cancer: a Cancer Research Campaign trial. *Br. J. Cancer*, **59**, 578-583.

WORLD HEALTH ORGANIZATION (1979). *WHO Handbook for Reporting Results of Cancer Treatment*. WHO offset publications No. 48, WHO, Geneva.

ZIGMOND, A.S. & SNAITH, R.R. (1983). The Hospital Anxiety and Depression scale. *Acta Psychiat. Scand.*, **67**, 361-370.